# Comprehensive Analysis of E2F Family Members in Human Gastric Cancer

**DOI:** 10.3389/fonc.2021.625257

**Published:** 2021-08-31

**Authors:** Shengbo Li, Xiaofan Yang, Wenqing Li, Zhenbing Chen

**Affiliations:** ^1^Department of Hand Surgery, Union Hospital, Tongji Medical College, Huazhong University of Science and Technology, Wuhan, China; ^2^Department of Hand and Foot Surgery, Huazhong University of Science and Technology Union Shenzhen Hospital, Shenzhen, China

**Keywords:** E2F, gastric cancer, comprehensive bioinformatics analysis, biomarkers, prognosis

## Abstract

Gastric cancer (GC) is the second most common cancer and the third most frequent cause of cancer-related deaths in China. E2Fs are a family of transcription factors reported to be involved in the tumor progression of various cancer types; however, the roles of individual E2Fs are still not known exactly in tumor progression of GC. In this study, we examined the expression of E2Fs to investigate their roles in tumor progression in GC patients using multiple databases, including ONCOMINE, GEPIA2, Kaplan-Meier plotter, cBioPortal, Metascape, LinkedOmics, GeneMANIA, STRING and UCSC Xena. We also performed real-time polymerase chain reaction (RT-PCR) to validate the expression levels of individual E2Fs in several GC cell lines. Our results demonstrated that the mRNA levels of *E2F1/2/3/5/8* were significantly higher both in GC tissues and cell lines. The expression levels of *E2F1* and *E2F4* were correlated with poor overall survival (OS), decreased post-progression survival (PPS), and decreased progression-free survival (FP) in patients with GC. However, overexpression of *E2F2*, *E2F5*, *E2F7* and *E2F8* is significantly associated with disease-free survival and overall survival in patients with GC. In addition, higher *E2F3* and *E2F6* mRNA expression was found to increase GC patients’ OS and PPS. 224 of 415 patients with STAD (54%) had gene mutations that were associated with longer disease-free survival (DFS) but not OS. Cell cycle pathway was closely associated with mRNA level of more than half of E2Fs (*E2F1/2/3/7/8*). There were close and complicated interactions among E2F family members. Finally, our results indicated the gene expressions of E2Fs had a positive relationship with its copy numbers. Taken together, *E2F1/2/3/5/8* can serve as biomarkers for GC patients with high prognostic value for OS of GC patients or therapeutic targets for GC.

## Introduction

Gastric cancer (GC) is the second most common cancer and the third most frequent cause of cancer-related deaths in China (1). Most stomach tumors are stomach adenocarcinoma (STAD) (90%–95%) (2). The high mortality rate of GC patients is often related to the difficulty of diagnosing GC, which is often due to its lack of early symptoms (3). Efforts have been made to explore the therapeutic targets for the treatment of GC, with some progress (4–7); however, some molecular characteristics of GC remain unknown. Elucidating the underlying mechanisms of the pathogenesis and etiology of GC would help to discover new diagnostic biomarkers and develop new treatments for GC.

E2Fs are a set of transcription factors encoded by a family of genes. Eight members (*E2F1–E2F8*) of the E2F family have been identified. It has been reported that E2Fs are primarily involved in the cell cycle regulation and DNA synthesis and are associated with various tumors. An increasing number of studies have found abnormal expression or activation of some E2Fs and have investigated their prognostic value in GC (8–13). Nevertheless, the exact role of each E2F member in development and progression of GC remains unknown.

Developments in sequencing technologies and multiple databases have made it possible to comprehensively analyze E2Fs. In the present study, data from several large public databases were used for comprehensive bioinformatics analyses of different E2Fs and their associations with clinical parameters in GC patients. Real-time polymerase chain reaction (RT-PCR) was performed to confirm the differentially expressed levels of E2Fs in two GC cell lines. Furthermore, we analyzed the functions of E2Fs in GC. Finally, we explored the potential drivers of the abnormal mRNA levels of E2Fs in GC.

## Materials and Methods

### ONCOMINE Analysis

ONCOMINE (http://www.oncomine.org) is an online cancer microarray database. The E2F mRNA levels were compared between the 20 cancer types and their normal controls using ONCOMINE (accessed between June and October 2020). The parameters used in this process were set as follows: *p* = 0.01, fold change = 1.5, gene rank = 10%, and data type = mRNA. Multiple testing correction was conducted using the false discovery rate (FDR) method.

### GEPIA2 Dataset

The GEPIA2 database (http://gepia2.cancer-pku.cn) is an updated version of GEPIA, containing RNA sequencing data of 9,736 tumors and 8,587 normal samples from The Cancer Genome Atlas (TCGA) and Genotype Tissue Expression (GTEx) projects (14). The GEPIA2 database (accessed between June and October 2020) was used to analyze the differential mRNA levels of E2Fs, for plot profiling, and to detect similar genes. Differences in the mRNA levels were compared by analysis of variance (ANOVA). Multiple testing correction was conducted using the FDR method, and the significance was set at *p*-value <0.01.

### Cell Culture and RT-PCR

The GC cell lines MGC-803 and SGC-7901 and the normal gastric epithelial cell line GES-1 were used. Cells were grown in RPMI 1640 medium (Gibco, Waltham, MA, USA) supplemented with 10% fetal bovine serum (FBS; Gibco) and 1% penicillin and streptomycin at 37°C with 5% CO2. Total RNA was isolated using an RNA extraction kit (#CW0581M, CWBIO, Tianjin, China) according to the manufacturer’s instructions. RNA was reverse transcribed into cDNA using a HiScript^®^ III RT SuperMix for qPCR (+gDNA wiper) kit (#R323-01, Vazyme, Nanjing, China). RT-PCR was conducted on the StepOnePlus™ platform (Applied Biosystems, Foster, CA, USA) using a ChamQ™ SYBR^®^ qPCR master mix kit (#Q311-02, Vazyme). Primer sequences are presented in **Table 1**. Relative gene expression levels were calculated using the 2^–ΔΔCt^ method and normalized to *ACTB*.

**Table 1:** Primer sequences of E2Fs for RT-PCR.

Gene	Forward primer	Reverse primer
*E2F1*	CCGTGGACTCTTCGGAGAACT	GGTTCTTGCTCCAGGCTGAGT
*E2F2*	TCGGTATGACACTTCGCTGGG	AACATTCCCCTGCCTACCCAC
*E2F3*	CCGCTTCCAAAGACTTGGCT	CATCGAAGAGATCGCTGATGCC
*E2F4*	GGACCCAACCCTTCTACCTCCT	CCGAGCTCATGCACTCTCGT
*E2F5*	GGGCTGCTCACTACCAAGTTC	CCAGCACCTACACCTTTCCAC
*E2F6*	AGCATTCAGGCCTTCCATGAAC	GCACTGTGATAGAGTCTTCTCTGG
*E2F7*	ACCCGACTGTCCCTCTTCATC	CAGAGCCAAGCTGGTCAGAAC
*E2F8*	CCTGAGATCCGCAACAGAGAT	AGATGTCATTATTCACAGCAGGG
*ACTB*	CAGCCTTCCTTCCTGGGCAT	GGGCAGTGATCTCCTTCTGCAT

### Kaplan-Meier Plotter

The Kaplan-Meier plotter (http://www.kmplot.com) is an online database that can assess the roles of 54,000 genes (coding for mRNAs, miRNAs, and proteins) in the survival of 21 cancer types, including breast (n = 6,234), ovarian (n = 2,190), lung (n = 3,452), and gastric (n = 1,440) cancers (15). In this study, the Kaplan-Meier plotter (accessed between June and October 2020) was used to analyze the effects of E2Fs mRNA levels on the overall survival (OS), post-progression survival (PPS), and first progression (FP) of GC patients. The JetSet best probe set was used as the probe-set option. Patients were split using the auto-selected best cutoff. The cutoff for significance was set to *p* < 0.05.

### cBioPortal

cBioPortal (http://www.cbioportal.org) is an online database that can conduct multidimensional cancer genomics studies (16). The STAD (The Cancer Genome Atlas, Firehose legacy) dataset (accessed between June and October 2020), containing 478 samples, was selected. The genomic profiles were composed of mutations, putative copy-number alterations from GISTIC, and mRNA expression z-scores (RNASeq V2 RSEM), and the z-score threshold was set at ±1.8. Samples with mRNA data (RNA Seq V2) (415) ware selected as patient/case set. The correlation of genetic mutations with OS and disease-free survival (DFS) of STAD patients was analyzed using the log-rank test with a significance threshold of *p* < 0.05. Co-expression analysis was conducted, using the cBioPortal’s online instruction as a reference.

### Functional Enrichment Analysis

Functional enrichment analysis of E2Fs and the genes similar to them was conducted using GEPIA2 (http://gepia2.cancer-pku.cn, accessed between June and October 2020) and Metascape (https://metascape.org) (accessed between June and October 2020). Metascape provides comprehensive gene list annotation and analysis resources (17). First, for each E2F family member, the top 30 similar genes that have a similar expression pattern in STAD were identified using GEPIA2 datasets. Metascape was used to perform Gene Ontology (GO) and Kyoto Encyclopedia of Genes and Genomes (KEGG) enrichment pathway analysis of the E2Fs and similar genes. The parameters were set as follows: *p* < 0.01, minimum count of 3, and enrichment factor > 1.5. A protein-protein interaction (PPI) network was created using BioGrid, InWeb_IM, and OmniPath. In addition, the molecular complex detection (MCODE) algorithm was utilized to analyze clusters of the PPI networks.

Furthermore, gene set enrichment analysis (GSEA) was also conducted using the online tool LinkedOmics (http://www.linkedomics.org/admin.php, accessed between June and October 2020). LinkedOmics is an open access portal that contains the multi-omics data of 32 TCGA Cancers (18). TCGA_STAD was selected as the interested cancer cohort, for which RNAseq datatype was selected as search dataset and target dataset. The interesting list was gsea_result_3583_1616921367.rnk. The interesting list contains 17608 unique entrezgene IDs. The expression dataset of 6843 genes related to the expression of E2Fs in 415 samples was used to perform GSEA using the “LinkInterpreter” module. The top-ranking enrichment term for each E2F factor was shown.

### Network Analysis

*E2F*-gene and E2F-protein interaction networks were constructed using GeneMANIA (https://genemania.org, accessed between June and October 2020) and STRING (https://string-db.org, accessed between June and October 2020). GeneMANIA provides user information about protein and genetic co-expression, co-localization, interactions, pathways, and shared protein domains of submitted genes (19). The data, including 325 co-expression datasets, four co-localization datasets, 10 genetic interaction datasets, six pathway datasets, 244 physical interactions datasets, 42 interaction prediction datasets, and two shared protein domain collections, were used to identify the interactions among the E2F genes. The STRING database collects and integrates all publicly available PPI data and predicts potential functions (20). STRING was used to perform a protein network interaction analysis among the E2Fs.

### UCSC Xena

UCSC Xena (https://xenabrowser.net) is a genome-related database that can be used as a visualization and analysis tool (21). Data on the relationship between the gene expression levels of E2Fs and their copy number segments were downloaded from the Genomic Data Commons (GDC) TCGA STAD data in the UCSC Xena browser (accessed between June and October 2020).

### Statistical Analysis

IBM SPSS 19.0 software was used for statistical analyses. Measurement data were analyzed using the Student’s *t*-test. The significance threshold of *p*-value was 0.05.

## Results

### Differential Expressions of E2Fs in GC

**Figure 1:**
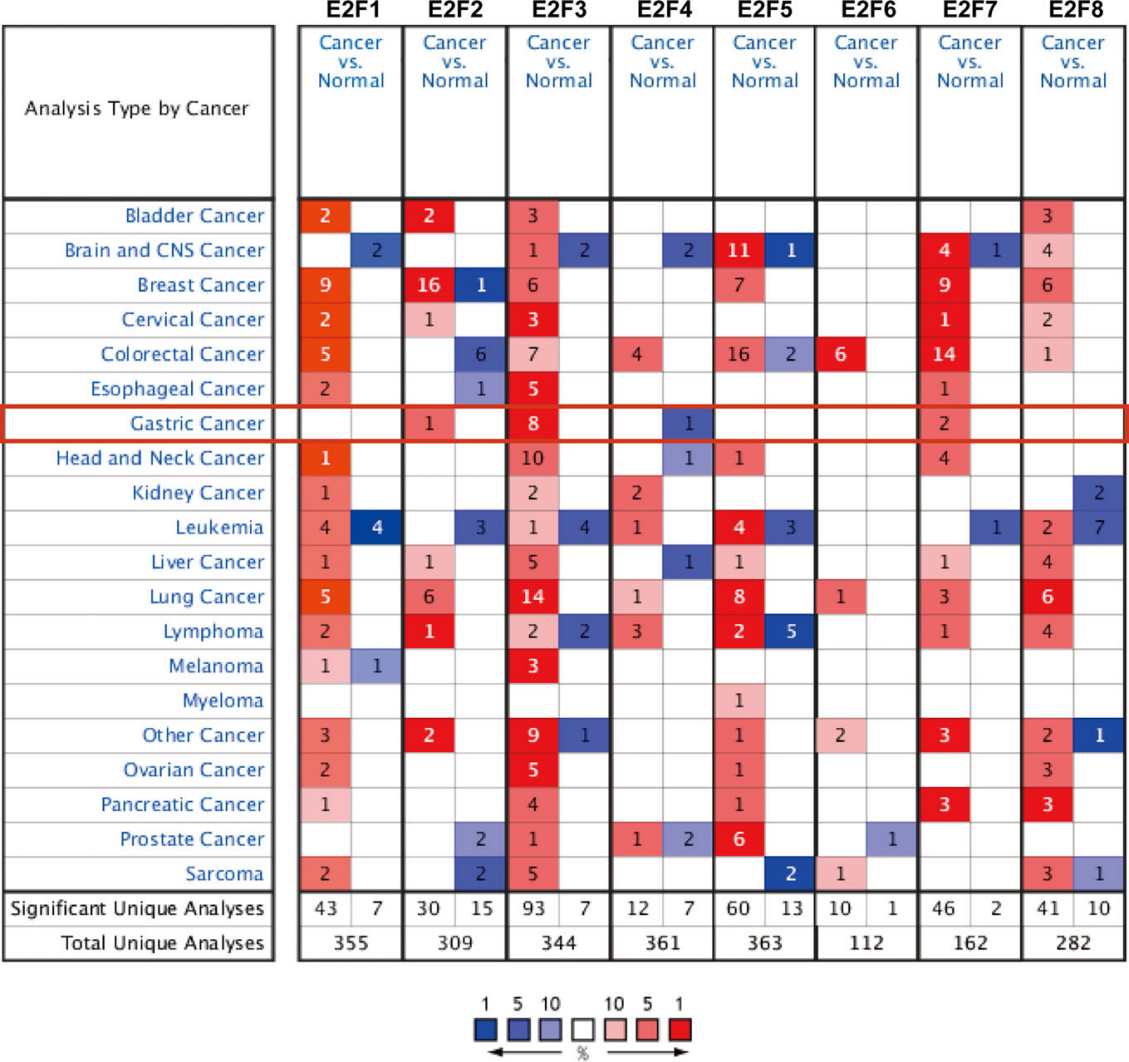
The mRNA expression levels of E2Fs in 20 different types of cancer diseases (ONCOMINE). Differences in the mRNA levels were compared by student’s *t*-test. The parameters were set as follows: *p* = 0.01, fold change = 1.5, gene rank = 10%, and data type = mRNA. Multiple testing correction was conducted using the false discovery rate (FDR) method.

The results obtained from the ONCOMINE database are presented in **Figure 1** and **Table 2**. FDR method was used to correct for multiple testing. Significantly higher mRNA expression of *E2F2/3/7* was found in GC tissues in multiple datasets (**Figure 1**). In DErrico’s Gastric dataset (22), *E2F2* was overexpressed in gastric mixed adenocarcinoma tissues compared with that in the normal controls, with a fold change of 2.256 (p = 8.84E-05). Significant upregulation of *E2F3* was also found in different GC tissues compared to that in normal tissues. In addition, Cui et al. have shown that *E2F3* is upregulated in GC (fold change = 1.556, *p* = 6.88E-07) compared to that in the normal controls (12). Wang et al. have also reported that *E2F3* is overexpressed in GC (fold change=2.261, *p*=1.99E-04) (13). Furthermore, *E2F7* was upregulated in GC (fold change = 1.977, *p* = 3.93E-05) and gastric intestinal-type adenocarcinoma (fold change = 3.234, *p* = 3.39E-07) (**Table 2**).

**Table 2:** Changes of E2Fs mRNA levels between different types of GC and normal gastric tissues (ONCOMINE).

	Types of gastric cancer	Fold change	P value	t-test	Ref	PMID
*E2F2*	Gastric Mixed Adenocarcinoma	2.256	8.84E-05	4.989	DErrico Gastric	19081245
*E2F3*	Diffuse Gastric Adenocarcinoma	1.74	4.08E-11	8.3	Cho Gastric	21447720
	Gastric Intestinal Type Adenocarcinoma	1.503	2.83E-04	3.918	Cho Gastric	21447720
	Gastric Mixed Adenocarcinoma	1.609	0.002	3.637	Cho Gastric	21447720
	Gastric Intestinal Type Adenocarcinoma	1.596	8.39E-13	8.635	Chen Gastric	12925757
	Gastric Cancer	1.556	6.88E-07	5.027	Cui Gastric	20965966
	Gastric Mixed Adenocarcinoma	2.862	4.79E-06	9.767	DErrico Gastric	19081245
	Gastric Intestinal Type Adenocarcinoma	2.374	1.50E-10	7.842	DErrico Gastric	19081245
	Gastric Cancer	2.261	1.99E-04	4.197	Wang Gastric	21132402
*E2F7*	Gastric Cancer	1.977	3.93E-05	4.059	Cui Gastric	20965966
	Gastric Intestinal Type Adenocarcinoma	3.234	3.39E-07	5.611	DErrico Gastric	19081245

**Figure 2:**
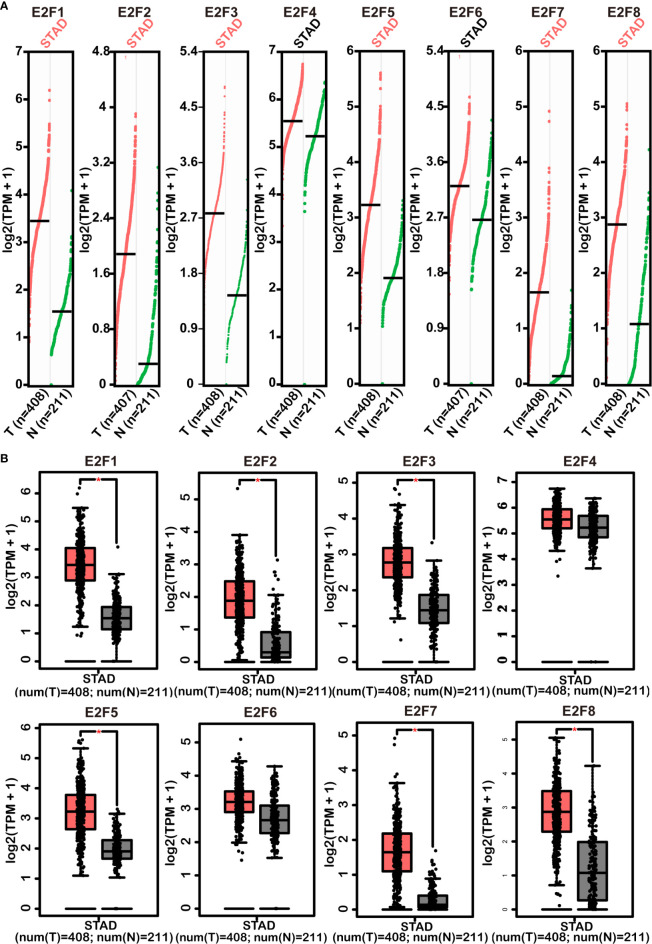
Comparisons of mRNA levels of E2Fs in STAD tissues with those in normal stomach tissues (GEPIA2). Scatter diagram **(A)** and box plot **(B)** represented differences of the mRNA level of E2Fs between STAD tissues and normal controls. Multiple testing correction was conducted using the false discovery rate (FDR) method. The parameters were set as follows: *p* = 0.01, |Log2FC| = 1, matched normal data = match TCGA normal and GTEx data. Red versus grey in **(B)** represents tumor tissues versus normal tissues. The pink STAD in **(A)** and * in **(B)** indicate that the differences are statistically significant. STAD, stomach adenocarcinoma; TPM, transcripts per million; T, tumor samples; N, normal samples.

**Figure 3:**
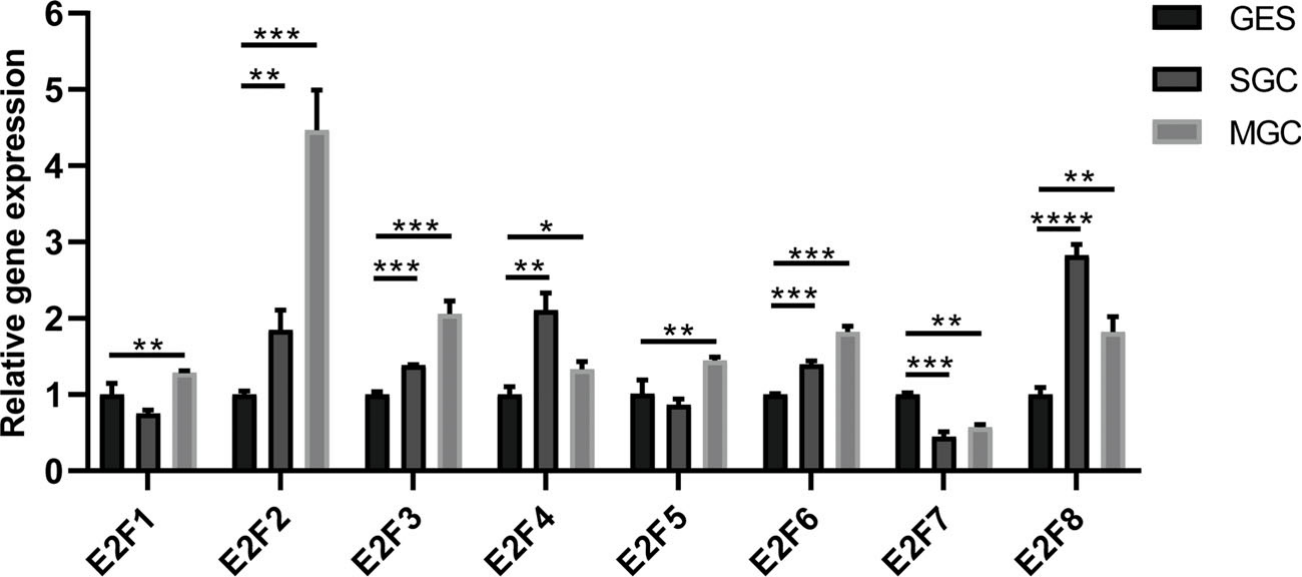
Differential expressions of E2Fs in GC cell lines and normal gastric epithelial cell line. MGC, gastric cancer cell line MGC-803; SGC, gastric cancer cell line SGC-7901; GES, normal gastric epithelial cell line GES-1. *p < 0.05, **p < 0.01, *** *p* < 0.001, and ****p < 0.0001.

The results from the GEPIA2 dataset indicated that higher mRNA levels of *E2F1/2/3/5/7/8* were observed in STAD tissues compared with those in the normal controls, whereas *E2F4/6* were not differentially transcribed in STAD tissues compared with those in the normal tissues (**Figure 2**). FDR method was used to correct for multiple testing. The results from RT-PCR indicated that all E2Fs, except *E2F7*, were overexpressed in GC cell lines (**Figure 3**).

### Prognostic Value of mRNA Expression of E2Fs in GC Patients

**Figure 4:**
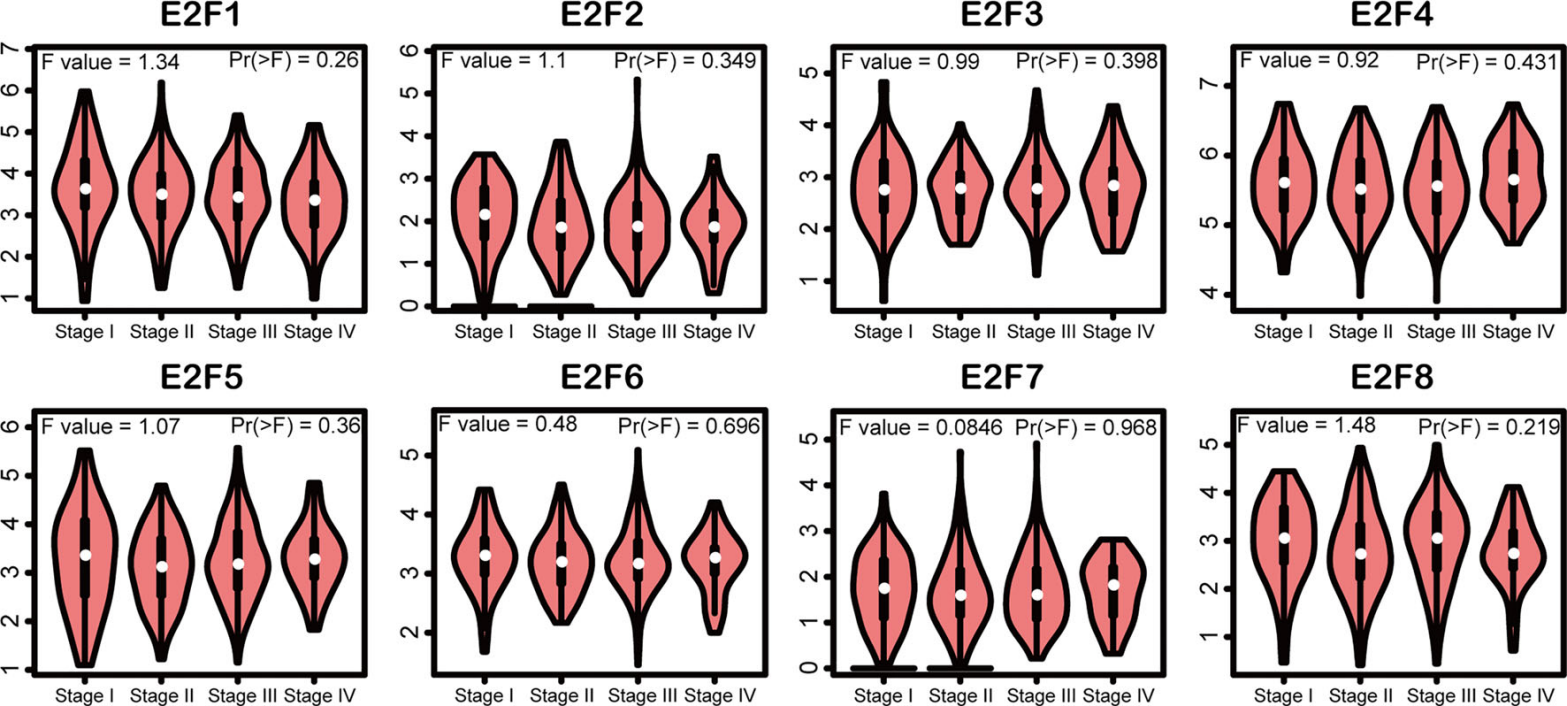
Association of mRNA expression of E2Fs with tumor stages of STAD patients (GEPIA2). Violin plots depicted the differences of mRNA level of E2Fs across STAD tumor stage. The statistical significance was examined using one-way analysis of variance (ANOVA). The significance threshold was set at *p* < 0.01.

**Figure 5:**
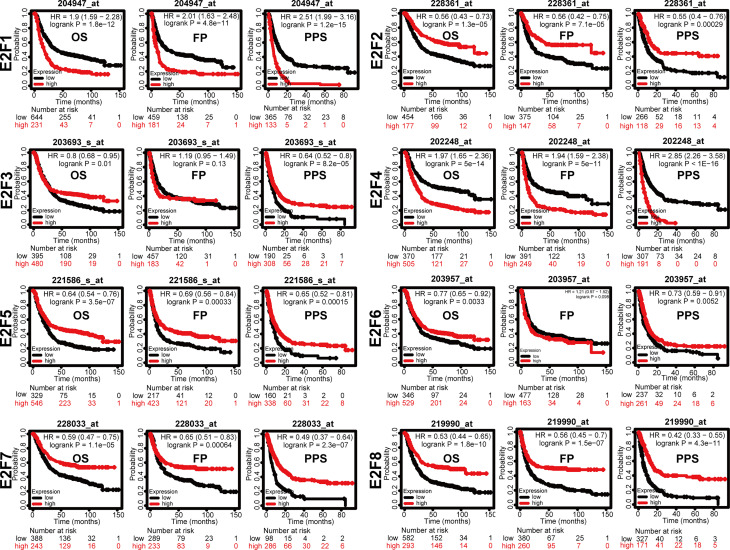
Prognostic value of mRNA expression of E2Fs in GC patients (Kaplan-Meier Plotter). Overall survival curve, progression-free survival, and post-progression survival curve of GC patients were plotted. The JetSet best probe set was used as the probe set option. Patients were split by the auto selected best cutoff. If the expression level was higher than the cutoff value, the OS curve was marked as high; if the expression level was lower than the cutoff value, the OS curve was marked as low. The cutoff for significance was set to *p* < 0.05. OS, overall survival; FP, first progression; PPS, post-progression survival.

We tried to elucidate the relationship between the mRNA levels of E2Fs and the STAD tumor stage. As shown in **Figure 4**, we unexpectedly found no significant associations between the mRNA levels of E2Fs and tumor stages (*p* > 0.01). We further found that the increased transcriptional levels of *E2F1* and *E2F4* were correlated with unfavorable OS, FP, and PPS (*p* < 0.05) in the GC patients (**Figure 5**). However, the increased transcriptional levels of *E2F2/5/7/8* favored the OS, FP, and PPS of GC patients (*p* < 0.05) (**Figure 5**). In addition, high transcriptional levels of *E2F3/6* significantly favored OS and PPS in GC patients (*p* < 0.05) (**Figure 5**).

### Gene Mutations of E2Fs and Their Significance in OS and DFS of STAD Patients

**Figure 6:**
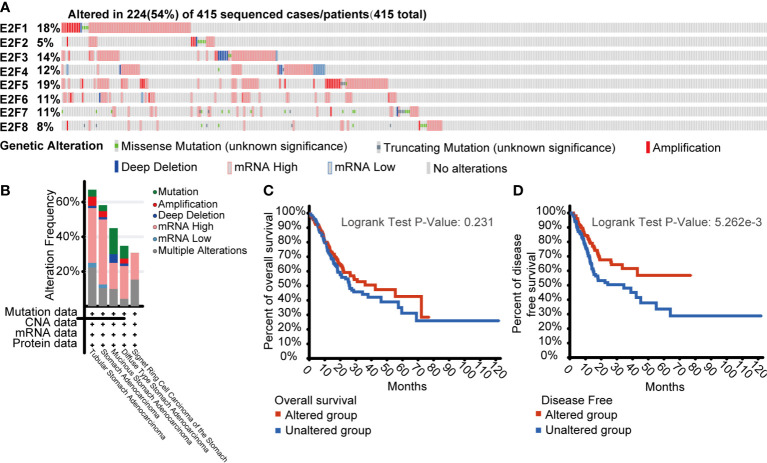
Gene mutations of E2Fs and their significance in overall survival (OS) and disease-free survival (DFS) of STAD patients (cBioPortal). **(A)** Analysis of gene mutations of E2Fs in GC. A total of 415 patients with STAD were analyzed, and gene mutations were found in 224 (54%) patients. *E2F5*, *E2F1*, *E2F3*, and *E2F4* had the highest gene mutation percentages (19%, 18%, 14%, and 12%, respectively). **(B)** Cancer types summary of mutations in E2Fs. Patients with tubular STAD were the most likely to have gene alterations of E2Fs (67.11% of 76 cases). **(C)** Kaplan-Meier OS curve by E2Fs gene mutations status. **(D)** Kaplan-Meier DFS curve by E2Fs gene mutations status.

To assess the gene mutations of E2Fs and their relevance to OS and DFS, we used the cBioPortal online tool for STAD (TCGA, Firehose Legacy; https://www.cbioportal.org). As shown in **Figure 6A**, a total of 415 patients with STAD were analyzed, and gene mutations were found in 224 (54%) patients. *E2F5*, *E2F1*, *E2F3*, and *E2F4* had the highest gene mutation percentages (19%, 18%, 14% and 12%, respectively). As shown in **Figure 6B**, patients with tubular STAD were the most likely to have gene alterations of E2Fs (67.11% of 76 cases). Furthermore, genetic alterations in E2Fs were associated with longer DFS (**Figure 6D**, *p* = 5.262E-3) but not OS (**Figure 6C**, *p* = 0.231) of STAD patients.

### Enrichment Analysis of E2Fs and the Genes Similar to Them in STAD Patients

**Figure 7:**
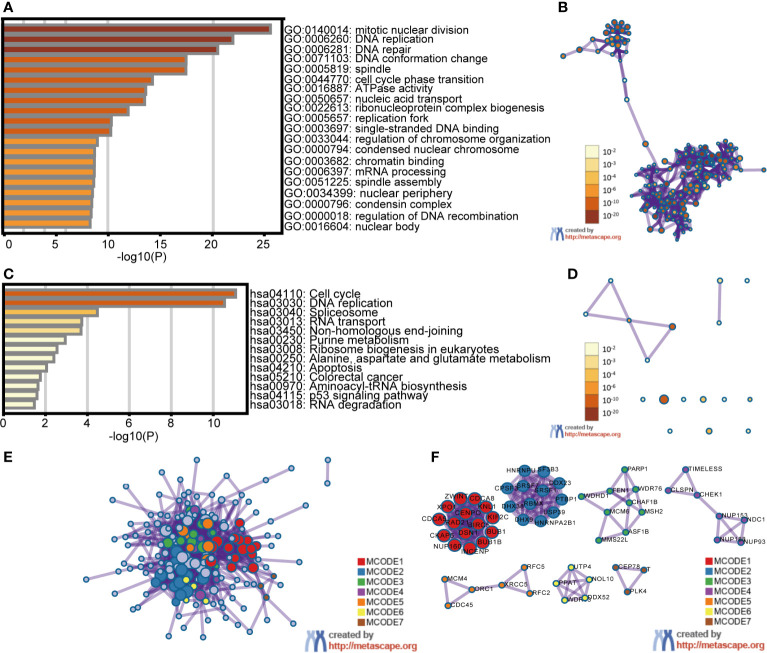
Enrichment analysis of E2Fs and the genes similar to them in STAD patients (Metascape). The top 30 similar genes of each E2F family member in STAD were identified using the GEPIA2 datasets. The Metascape was used to perform Gene Ontology (GO) and Kyoto Encyclopedia of Genes and Genomes (KEGG) enrichment pathway analysis of the E2Fs and similar genes. A subset of enriched terms was selected and rendered as network plots to further reveal the relationships between the enriched terms. A Protein-protein interaction (PPI) network was created using BioGrid, InWeb_IM, and OmniPath. In addition, the molecular complex detection (MCODE) algorithm was utilized to analyze clusters of the PPI network. **(A)** Heatmap of GO enriched terms colored by *p*-value. **(B)** Network of GO enriched terms colored by *p*-value, where terms containing more genes tend to have a more significant *p*-value. **(C)** Heatmap of KEGG enriched terms colored by *p*-value. **(D)** Network of KEGG enriched terms colored by *p*-value, where terms containing more genes tend to have a more significant *p*-value. **(E)** PPI network of genes similar to E2Fs. **(F)** MCODE components identified in genes similar to E2Fs.

First, the top 30 similar genes that have a similar expression pattern in STAD were identified using GEPIA2 datasets. The GO and KEGG enrichment pathway analyses of E2Fs and their similar genes were performed using Metascape. The results of GO and KEGG analyses are displayed in **Figure 7** and **Tables 3**, **4**. Eleven biological process (BP) items, six cellular component (CC) items, and three molecular function items made up the top 20 GO enriched list (**Figures 7A, B** and **Table 3**). BPs such as mitotic nuclear division, DNA replication, DNA repair, DNA conformation change, and cell cycle phase transition were significantly regulated by these genes. CCs, including spindles, replication forks, condensed nuclear chromosomes, nuclear peripheries, condensin complexes, nuclear bodies, were remarkably associated with E2Fs and the similar genes. Additionally, the MFs associated with these genes were ATPase activity, single-stranded DNA binding, and chromatin binding. KEGG pathway analysis revealed the involvement of E2Fs in pathways such as the cell cycle, DNA replication, spliceosome, and RNA transport in STAD (**Figures 7C, D** and **Table 4**). PPI enrichment analysis was performed, and significant modules were identified (**Figures 7E, F**). Seven MCODE components were extracted, which were mainly associated with chromosomes, centromeric regions, chromosomal regions, and kinetochores.

**Table 3:** GO function enrichment analysis of E2Fs and their similar genes in STAD (Metascape).

GO	Category	Description	Count	%	Log10(P)	Log10(q)
GO:0140014	GO Biological Processes	mitotic nuclear division	32	14.35	-25.43	-21.08
GO:0006260	GO Biological Processes	DNA replication	29	13.00	-21.86	-18.17
GO:0006281	GO Biological Processes	DNA repair	37	16.59	-20.43	-17.03
GO:0071103	GO Biological Processes	DNA conformation change	27	12.11	-17.42	-14.20
GO:0044770	GO Biological Processes	cell cycle phase transition	32	14.35	-14.24	-11.13
GO:0050657	GO Biological Processes	nucleic acid transport	19	8.52	-13.49	-10.48
GO:0022613	GO Biological Processes	ribonucleoprotein complex biogenesis	25	11.21	-11.92	-9.07
GO:0033044	GO Biological Processes	regulation of chromosome organization	19	8.52	-9.00	-6.42
GO:0006397	GO Biological Processes	mRNA processing	22	9.87	-8.71	-6.18
GO:0051225	GO Biological Processes	spindle assembly	12	5.38	-8.66	-6.13
GO:0000018	GO Biological Processes	regulation of DNA recombination	11	4.93	-8.42	-5.93
GO:0005819	GO Cellular Components	spindle	28	12.56	-17.40	-14.20
GO:0005657	GO Cellular Components	replication fork	11	4.93	-10.33	-7.60
GO:0000794	GO Cellular Components	condensed nuclear chromosome	11	4.93	-8.74	-6.19
GO:0034399	GO Cellular Components	nuclear periphery	12	5.38	-8.54	-6.02
GO:0000796	GO Cellular Components	condensin complex	5	2.24	-8.47	-5.96
GO:0016604	GO Cellular Components	nuclear body	27	12.11	-8.32	-5.85
GO:0016887	GO Molecular Functions	ATPase activity	25	11.21	-13.60	-10.55
GO:0003697	GO Molecular Functions	single-stranded DNA binding	13	5.83	-10.27	-7.56
GO:0003682	GO Molecular Functions	chromatin binding	23	10.31	-8.72	-6.18

**Table 4:** KEGG enrichment analyses of E2Fs and their similar genes in STAD (Metascape).

GO	Category	Description	Count	%	Log10(P)	Log10(q)
hsa04110	KEGG Pathway	Cell cycle	14	6.28	-11.07	-8.37
hsa03030	KEGG Pathway	DNA replication	9	4.04	-10.54	-8.14
hsa03040	KEGG Pathway	Spliceosome	8	3.59	-4.47	-2.38
hsa03013	KEGG Pathway	RNA transport	8	3.59	-3.73	-1.78
hsa03450	KEGG Pathway	Non-homologous end-joining	3	1.35	-3.69	-1.78
hsa00230	KEGG Pathway	Purine metabolism	7	3.14	-2.94	-1.09
hsa03008	KEGG Pathway	Ribosome biogenesis in eukaryotes	5	2.24	-2.55	-0.81
hsa00250	KEGG Pathway	Alanine, aspartate and glutamate metabolism	3	1.35	-2.40	-0.71
hsa04210	KEGG Pathway	Apoptosis	5	2.24	-2.05	-0.43
hsa05210	KEGG Pathway	Colorectal cancer	3	1.35	-1.75	-0.17
hsa00970	KEGG Pathway	Aminoacyl-tRNA biosynthesis	3	1.35	-1.64	-0.10
hsa04115	KEGG Pathway	p53 signaling pathway	3	1.35	-1.59	-0.08
hsa03018	KEGG Pathway	RNA degradation	3	1.35	-1.47	-0.01

**Figure 8:**
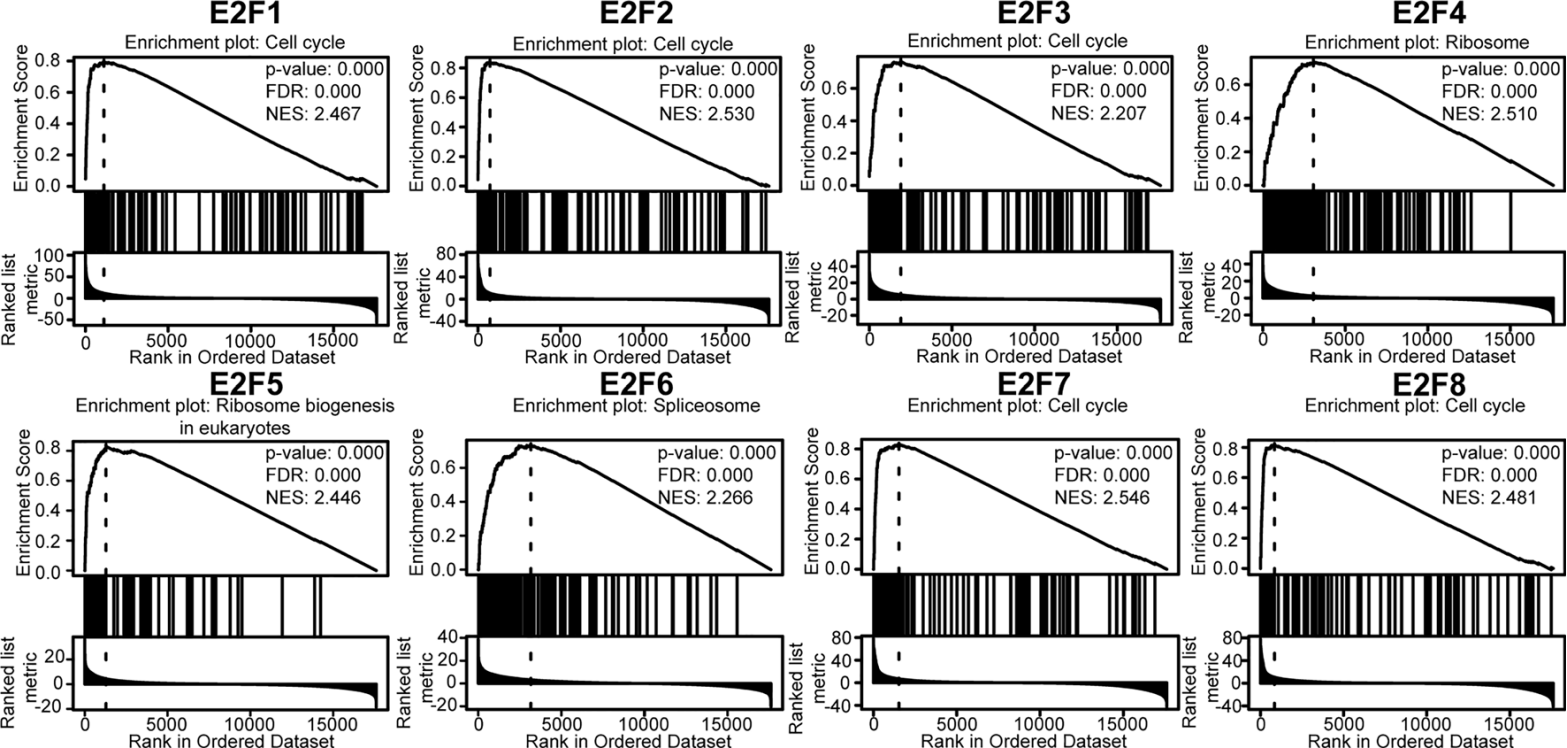
Gene set enrichment analysis (GSEA) of E2Fs (LinkedOmics). TCGA_STAD was selected as the interested cancer cohort, for which RNAseq datatype was selected as search dataset and target dataset. The interesting list was gsea_result_3583_1616921367.rnk. The interesting list contains 17608 unique entrezgene IDs. The expression dataset of 6843 genes related to the expression of E2Fs in 415 samples was used to perform GSEA using the “LinkInterpreter” module. The parameters were set as follows: enrichment analysis = KEGG pathway; rank criteria = FDR; minimum Number of Genes (Size) = 3; simulations = 500; *p* = 0.05; FDR < 0.25, |NES| > 1. The top-ranking enrichment term for each E2F factor was shown. TCGA, the cancer genome atlas; FDR, false discovery rate; NES, normalized enrichment score.

Furthermore, GSEA of the E2Fs at the gene level was performed using the online tool LinkedOmics. The GSEA results showed that the mRNA expressions of *E2F1/2/3/7/8* were closely associated with the cell cycle pathway (**Figure 8**). GSEA of *E2F4* revealed significant enrichment in the ribosome. GSEA of *E2F5* revealed a significant enrichment in ribosome biogenesis in eukaryotes, and GSEA of *E2F6* revealed a significant enrichment in the spliceosome (**Figure 8**).

### Co-expression and Interaction Analysis of E2Fs at Gene and Protein Levels in STAD Patients

**Figure 9:**
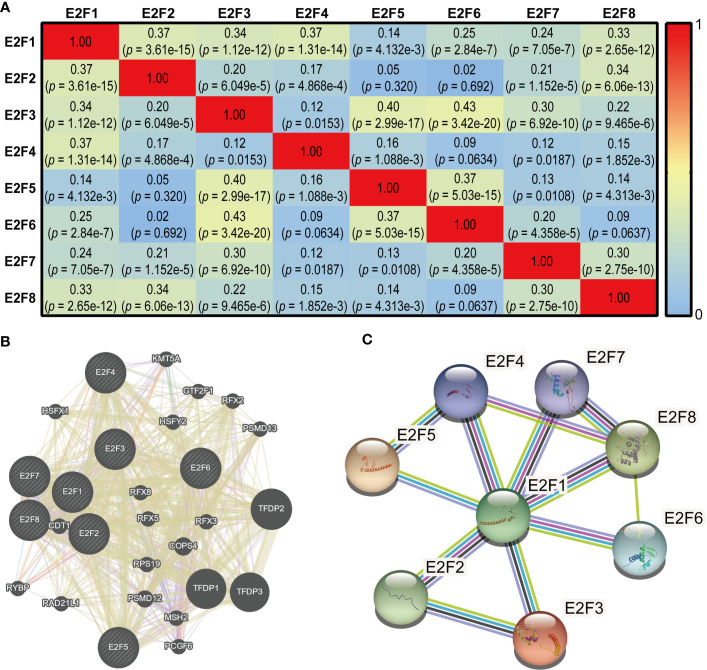
Co-expression and interaction analysis of E2Fs at gene and protein levels in STAD patients (cBioPortal, GeneMANIA and STRING). **(A)** Pearson correlation analysis of expression of E2Fs predicted by the cBioPortal. **(B)** Gene-gene interaction network among E2Fs predicted by the GeneMANIA. **(C)** Protein-protein interaction network among E2Fs predicted by the STRING.

As shown in **Figure 9A**, there were significant and moderate positive correlations between *E2F1* and *E2F2*, *E2F3*, *E2F4*, and *E2F8*; *E2F2* with *E2F1* and *E2F8*; *E2F3* with *E2F1*, *E2F5*, *E2F6*, and *E2F7*; *E2F4* with *E2F1*; *E2F5* with *E2F3* and *E2F6*; *E2F6* with *E2F3* and *E2F5*; *E2F7* with *E2F3* and *E2F8*; and *E2F8* with *E2F1*, *E2F2*, and *E2F7*. The correlations remained significant after Bonferroni correction (adjusted *p* = 0.0018). As shown in **Figure 9B**, the results of correlation analysis of the E2Fs at the gene level indicated that 28 genes, including *TFDP1*, *TFDP2* and *TFDP3*, were enriched in this network based on their functions related to co-expression, co-localization, genetic interactions, pathways, physical interactions, and shared protein domains. STRING was used to explore the potential interactions among the E2Fs at the protein level. As shown in **Figure 9C**, eight nodes and 12 edges were found in the PPI network.

### Correlations of Gene Copy Numbers With mRNA Levels of E2Fs in STAD

**Figure 10:**
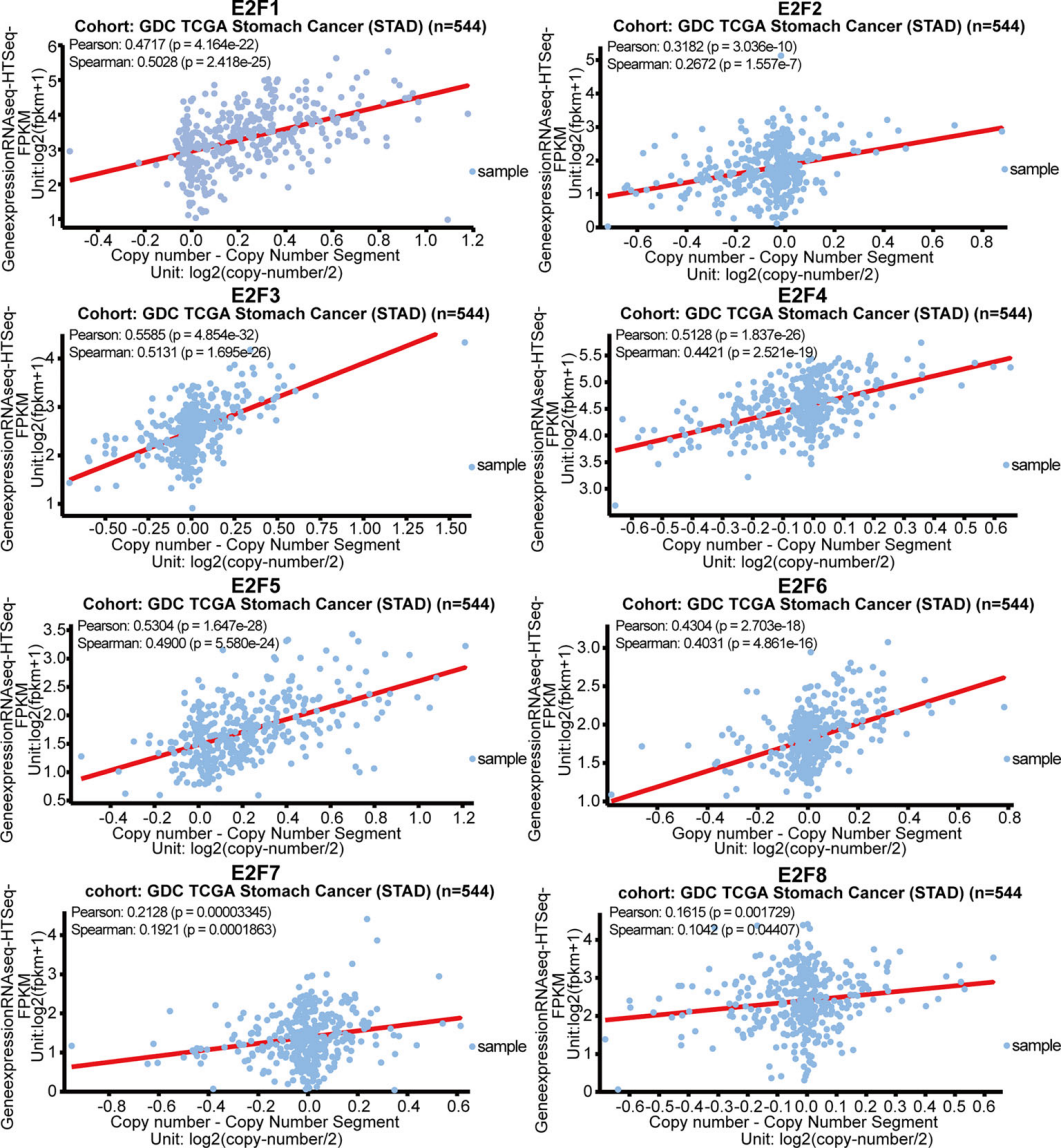
Correlations of gene copy numbers with mRNA levels of E2Fs in STAD (UCSC Xena).

We further explored the potential drivers of the elevated mRNA levels of the E2Fs in STAD. We sought to determine whether there was any correlation between the gene expression levels of E2Fs and the copy number segments from the GDC TCGA STAD data obtained using the UCSC Xena browser (https://xenabrowser.net). As indicated in **Figure 10**, the gene expression of all E2Fs had a positive and significant relationship with their copy numbers.

## Discussion

E2Fs have been shown to be associated with many tumors (23–26). As for GC, the vast majority of studies have focused on the roles of one particular member or some members from among the E2Fs, and there have been few comprehensive analyses of the expression of all E2Fs and their associations with clinical parameters in GC patients. Manicum et al. (27) reported associations between *E2F1/2/3/4/5/6/7* mRNA levels and OS in GC patients. Liu et al. (28) reported the prognostic value of the expression of E2Fs and its association with clinical parameters in GC patients using publicly available databases. In the present study, we confirmed the abnormal expression of E2Fs in GC using several large public databases. We also confirmed the differential expression levels of E2Fs in two GC cell lines and a normal gastric epithelial cell line by RT-PCR. Our results demonstrated that the mRNA levels of *E2F1/2/3/5/8* were significantly overexpressed in both GC tissues and two GC cell lines. In addition, we investigated the genetic alterations and their prognostic value in GC patients in detail and found genetic alterations in E2Fs was relevant to longer DFS but not OS. Furthermore, the functional enrichment analyses and interaction analyses were conducted, and the results of GSEA analyses revealed that cell cycle pathway was closely associated with mRNA level of more than half of E2Fs. Finally, we investigated the potential drivers of the abnormal E2FS mRNA levels in GC and found that the expressions of all E2Fs had a positive and significant relationship with the DNA copy numbers. So, we demonstrated the significance of E2Fs in GC from different perspectives and suggested that *E2F1/2/3/5/8* could serve as potential biomarkers for GC patients with high prognostic value for OS of GC patients or therapeutic targets for GC.

Biomarkers have been reported to exhibit tissue-specific expression (29). Our results from the ONCOMINE database indicated that E2F mRNA levels are elevated to various degrees in most cancers compared with their levels in normal tissues. To the best of our knowledge, pan-cancer analysis of E2Fs has not been previously reported. A comprehensive pan-cancer analysis of E2Fs would provide a deeper understanding of the nature of E2Fs dysregulation in cancer.

The mRNA levels of *E2F2/3/7* were increased in GC tissues in both the ONCOMINE and GEPIA2 datasets. The results from RT-PCR indicated that all E2Fs, except *E2F7*, were overexpressed at different levels in the GC cell lines, which might be due to cell line heterogeneity. Given that *E2F1* is a transcriptional activator and *E2F4* is a repressor, typically representing opposing activities, it was interesting to find that increased expression of both *E2F1* and *E2F4* was correlated with poor survival in GC patients. As a family of transcription factors, E2Fs play important roles in GC by modulating the transcription of specific target genes, and their regulatory activity and biological effects can be reflected by their target gene expression. It has been reported that high *E2F1* or *E2F4* activity in liposarcoma patients is associated with unfavorable prognosis, with the core target gene sets of *E2F1* containing 116 genes and the core target gene sets of *E2F4* containing 199 genes, among which only 21 are shared (30). As we have shown, GSEA indicated that *E2F1* was mainly involved in the cell cycle pathway; *E2F4*, however, was mainly involved in ribosome pathway. Thus, we reasoned that a similar situation might occur in GC, where *E2F1* and *E2F4* mainly exert effects on different target gene sets and only share a small fraction of common target genes. The systematic identification of E2F target gene sets in GC will further improve our understanding of the mechanisms behind the prognostic value of E2Fs in GC.

Genetic mutations have been reported to correlate with the pathogenesis and prognosis of various types of tumors (31–33); therefore, we further explored genetic mutations in E2Fs for GC, based on cBioPortal. We found that E2Fs had a relatively high mutation rate (54%) in GC patients, and the genetic mutations in E2Fs were associated with longer DFS but not OS. Genetic mutations in E2Fs appeared to have a protective role against the progression of GC. The clinical implications of this finding deserve further studies. We also performed enrichment analysis for E2Fs and 30 neighboring genes. GSEA analysis revealed that the cell cycle pathway was closely associated with the mRNA level of more than half of the E2Fs (*E2F1/2/3/7/8*). These results also emphasized the conserved functions of the E2Fs. The results of co-expression and interaction analyses revealed close and complicated associations among the E2Fs. It has been reported that DNA copy number is positively associated with the expression levels for 98.9% of all the abundantly expressed human genes, indicating global gene dosage sensitivity (34). Therefore, we investigated the potential drivers of expression dysregulation of E2Fs in GC and found that the gene expression of all E2Fs had a positive and significant relationship with their DNA copy numbers, and this extends the results of the E2F gene expression studies and can guide further efforts in identifying the potential mechanisms of pathogenesis and treatments for GC.

*E2F1*, the most investigated member of E2Fs, has been confirmed to have prognostic value in many tumors, such as hepatocellular carcinoma (35), breast cancer (36), and pancreatic ductal adenocarcinoma (37), and spinal osteosarcoma (38). In GC, upregulated *E2F1* expression, targeting the TINCR/STAU1/CDKN2B signaling axis, was positively associated with poor prognosis due to its association with advanced stage and larger tumor size (39). *E2F1* increased the expression of the miR-106b-25 cluster, leading to the impairment of TGFβ-dependent cell-cycle arrest and resistance to TGFβ-dependent apoptosis (40). Lin et al. (41) reported that circCYFIP2, serving as an oncogenic circRNA, promoted GC progression by regulating the miR-1205/*E2F1* axis. Simultaneous silencing of *E2F1* inhibits GC progression (42). However, another study reported that *E2F1* overexpression inhibited GC progression *in vitro* (43). In the present study, *E2F1* was significantly overexpressed in GC tissues and in MGC-803 cells. Overexpression of *E2F1*, mainly related to the cell cycle pathway, was associated with poor OS, FP, and PPS in GC patients.

The role of *E2F2* in GC has been less frequently studied. Recently, it has been reported that downregulation of miR-31, one of the direct target genes of *E2F2*, is related to poor prognosis in GC patients (44). Furthermore, another study found that miR-26a showed low expression in cisplatin-resistant GC cells; and the knockdown of *E2F2*, a direct target gene of miR-26a, sensitized GC cells to cisplatin-based chemotherapies. The results from our study indicated that *E2F2* overexpression was observed in GC tissues and GC cell lines. The increased *E2F2* mRNA level favored OS, FP, and PPS in GC patients.

A previous study has shown that upregulated *E2F3* expression in GC might imply poor prognosis. MiR-152, which targets the 3′-UTR of *E2F3* and reduces its expression, regulates polo-like kinase 1 (PLK1) mediated protein kinase B and extracellular signal-regulated kinase signals, and modulates GC metastasis (45). *E2F3* might have a pro-oncogenic effect on GC metastasis and progression by regulating the miR-125a/DKK3 axis (46). In addition, by targeting *E2F3*, miR-564 acts a tumor suppressor in GC (47). The colorectal neoplasia differentially expressed (CRNDE) gene was a cancer-promoting lncRNA in GC; by competitive molecular sponging of miR145, CRNDE strongly stimulated the expression of *E2F3* (48). By interacting with lncRNA MEG3 and decreasing *E2F3* expression, miR-141 inhibits GC proliferation (49). The miR-449a/*E2F3* axis is involved in the biological processes of proliferation and apoptosis in GC (50). In our current analysis, *E2F3* was upregulated in GC patients and GC cell lines and had a favorable effect on OS and PPS.

In earlier investigations, pRb2/p130 negatively regulated the cell cycle by interacting with the *E2F4* and *E2F5*, thus playing oncosuppressive roles (51). Cyclins interact with some transcription factors, such as *E2F4*, *SIN3A*, *NFYA*, and *FOXM1*, while overexpression of cyclins is correlated with unfavorable prognosis in GC patients (52). In the progression of multiple GCs, mutations of *E2F4* are early events that occur even in the intestinal metaplastic mucosa (53). Knockdown of *RAD51* improved the effects of chemotherapy combined with PCI-24781 and cis-diamminedichloroplatinum, and during this process, the interaction of *E2F4* with the *RAD51* promoter had a major effect (54). In gastric adenocarcinoma which has frequent microsatellite instability, mutations of *E2F4* are integral multiples of three nucleotides lost or gained (55). In the current study, there was no significant difference in *E2F4* mRNA levels between GC tissues and normal control in the GEPIA2 database. However, *E2F4* was upregulated in the GC cell lines. GC patients with increased *E2F4* mRNA levels had poor OS, FP and PPS.

Similar to *E2F2*, there have been relatively few reports on *E2F5* in GC. It has been reported that miRNA-34a, by targeting *E2F5*, increases the sensitivity of GC cells to paclitaxel (56). Another study indicated that miR-106b promotes the cell cycling of GC cells by regulating p21 and *E2F5* (57). In the current study, *E2F5* mRNA levels were significantly higher in GC tissues and GC cell lines, which markedly favored OS, FP, and PPS in GC patients.

A relatively high *E2F6* mRNA level has been found in gastric adenocarcinoma with no lymph node metastasis, and low expression of *E2F6* in gastric adenocarcinoma could be considered an aggressive phenotype (58). It has also been reported that downregulation of lncRNA CASC2 mediated by *E2F6* predicts worse outcomes and facilitates cancer progression in GC patients (59). Induction of miR-31 decreases the expression of *E2F6* and *SMUG1*, improving GC cell sensitivity to 5-fluorouracil and inhibiting GC cell migration and invasion (60). We found that *E2F6* was overexpressed in GC patients according to the GEPIA2 datasets and GC cell lines and that high *E2F6* mRNA levels favored OS and PPS in GC patients.

*E2F7* and *E2F8* have been reported to be involved in several malignancies such as pancreatic cancer (61), gallbladder cancer (62), colorectal cancer (63, 64), cervical cancer (65), breast cancer (66) and lung cancer (67, 68). However, the involvement of *E2F7* and *E2F8* in GC has yet not been investigated. Our results indicated that *E2F7* mRNA levels were high in GC tissues. However, *E2F7* was downregulated in MGC-803 and SGC-7901 cells compared with its expression in GES-1 cells, and this might be due to cell line heterogeneity. *E2F7* and *E2F8* expression was not associated with tumor stage in patients with GC. The increased mRNA levels of *E2F7* and *E2F8*, mainly related to the cell cycle pathway, were correlated with favorable OS, FP and PPS in GC patients.

Our results indicated that there were no significant associations between the mRNA levels of E2Fs and GC stages, indicating that mRNA levels of E2Fs might reflect tumor burden and that E2Fs might participate in all stages of GC. Also, it is well-known that GC is of heterogeneity (69). Different GC subtypes have various biologic characteristics. It would be beneficial to evaluate the particular express pattern of E2Fs of different subtypes of GC. A randomized controlled trial with large sample size would be helpful to further validate our results and to further our investigation.

Validating with three cell lines instead of GC patient samples is one of the limitations. It remains uncertain how well cell lines reflect the biological characteristics of tumors. Systematic differences between cell lines and human cancers may be due to many factors such as culture conditions, clonal selection, and genomic instability (70). So, despite these promising results and their clinical implications, we should acknowledge the limitations, as well as directions for future research.

## Conclusions

In summary, our results indicated that the mRNA levels of *E2F1/2/3/5/8* were significantly increased in both GC tissues and cell lines compared with those in the control samples, which mainly resulted from gene amplification and were associated with the clinical outcomes of GC patients. Genetic alterations in E2Fs were associated with longer DFS but not OS in STAD patients. These results suggest that *E2F1/2/3/5/8* could serve as potential biomarkers of prognostic significance in GC. These results may help to better understand the mechanisms of GC and the development of E2Fs-mediated drugs for GC treatment. However, further investigations are warranted to examine our results and the clinical implications of E2Fs in cancer.

## Data Availability Statement

The datasets presented in this study can be found in online repositories. The names of the repository/repositories and accession number(s) can be found in the article/supplementary material.

## Author Contributions 

SL designed the research, conducted the experiments, performed statistical analyses and drafted the manuscript. XY and WL revised the draft. ZC provided administrative/technical/material assistances and supervised the study. All authors contributed to the article and approved the submitted version.

## Funding

This work was supported by the National Natural Science Foundation of China (Grant No. 81772094, 81974289).

## Conflict of Interest

The authors declare that the research was conducted in the absence of any commercial or financial relationships that could be construed as a potential conflict of interest.

## Publisher’s Note

All claims expressed in this article are solely those of the authors and do not necessarily represent those of their affiliated organizations, or those of the publisher, the editors and the reviewers. Any product that may be evaluated in this article, or claim that may be made by its manufacturer, is not guaranteed or endorsed by the publisher.
